# Resveratrol Treatment in Human *Parkin*-Mutant Fibroblasts Modulates cAMP and Calcium Homeostasis Regulating the Expression of Mitochondria-Associated Membranes Resident Proteins

**DOI:** 10.3390/biom11101511

**Published:** 2021-10-14

**Authors:** Anna Signorile, Anna Ferretta, Consiglia Pacelli, Nazzareno Capitanio, Paola Tanzarella, Maria Laura Matrella, Alessio Valletti, Domenico De Rasmo, Tiziana Cocco

**Affiliations:** 1 Department of Basic Medical Sciences, Neurosciences and Sense Organs, University of Bari “Aldo Moro”, 70124 Bari, Italy; anna.signorile@uniba.it (A.S.); anna.ferretta@uniba.it (A.F.); tanzarellapaola87@gmail.com (P.T.); maria.matrella@uniba.it (M.L.M.); alessio.valletti@uniba.it (A.V.); 2 Department of Clinical and Experimental Medicine, University of Foggia, 71122 Foggia, Italy; consiglia.pacelli@unifg.it (C.P.); nazzareno.capitanio@unifg.it (N.C.); 3 Institute of Biomembranes, Bioenergetics and Molecular Biotechnologies, Italian National Research Council (CNR), 70126 Bari, Italy

**Keywords:** resveratrol, parkin, mitochondria, cAMP, calcium (Ca^2+^), endoplasmic reticulum (ER)

## Abstract

Parkin plays an important role in ensuring efficient mitochondrial function and calcium homeostasis. *Parkin*-mutant human fibroblasts, with defective oxidative phosphorylation activity, showed high basal cAMP level likely ascribed to increased activity/expression of soluble adenylyl cyclase and/or low expression/activity of the phosphodiesterase isoform 4 and to a higher Ca^2+^ level. Overall, these findings support the existence, in *parkin*-mutant fibroblasts, of an abnormal Ca^2+^ and cAMP homeostasis in mitochondria. In our previous studies resveratrol treatment of *parkin*-mutant fibroblasts induced a partial rescue of mitochondrial functions associated with stimulation of the AMPK/SIRT1/PGC-1α pathway. In this study we provide additional evidence of the potential beneficial effects of resveratrol inducing an increase in the pre-existing high Ca^2+^ level and remodulation of the cAMP homeostasis in *parkin*-mutant fibroblasts. Consistently, we report in these fibroblasts higher expression of proteins implicated in the tethering of ER and mitochondrial contact sites along with their renormalization after resveratrol treatment. On this basis we hypothesize that resveratrol-mediated enhancement of the Ca^2+^ level, fine-tuned by the ER–mitochondria Ca^2+^ crosstalk, might modulate the pAMPK/AMPK pathway in *parkin*-mutant fibroblasts.

## 1. Introduction

*PARK2* and *PARK6*, which encode for parkin and PINK1 respectively, are genes responsible of the onset of familial Parkinson’s disease (PD) a progressive degenerative disorder of the central nervous system characterized by dopaminergic neurodegeneration in the substantia nigra pars compacta. Several studies in PD models proved hallmarked dysfunctions of mitochondria, in particular, defect of the respiratory chain complex I, which plays a central role in mitochondrial oxidative phosphorylation (OXPHOS) efficiency and capacity [1], depletion of ATP production, increased reactive oxygen species (ROS) and oxidative stress, anomalies in mitochondrial dynamics, trafficking and turnover, dysregulation in calcium homeostasis, and protein misfolding and aggregation [2,3,4,5,6,7,8,9,10].

Parkin, together with PINK1, is involved in one of the mitochondrial quality control pathways in the cells that identifies impaired mitochondria and selectively primes their elimination by mitophagy [11,12]. In particular, loss of mitochondrial membrane potential in damaged mitochondria induces the accumulation of PINK1, a serine/threonine kinase, on the outer mitochondrial membrane surface. Subsequently, PINK1 phosphorylates parkin, which translocates to damaged mitochondria mediating the selective removal of the damaged organelle, after ubiquitination of mitochondrial proteins [13,14,15]. Studies, in vitro and in vivo, on *parkin*-null models clearly indicate a role of parkin in the preservation of a functional mitochondrial compartment. Indeed, an altered mitochondrial respiration and morphology, decrease of mitochondrial ATP production and a higher susceptibility to neurotoxin 1-methyl-4-phenylpyridinium ion (MPP^+^) have been observed in parkin KO models [16,17,18,19,20]. Accordingly, mitochondrial impairment has been repeatedly observed in *parkin*-mutant non-neuronal cell-type-like fibroblasts [4,5,21,22,23]. 

Along with this line of evidence, we showed in earlier studies that primary *parkin*-mutant fibroblasts, carrying different mutations in the *PARK2* gene, displayed severe ultrastructural abnormalities, mainly in mitochondria [4,24], altered expression of proteins involved in structural dynamics of cytoskeleton, oxidative stress response, Ca^2+^ homeostasis, and RNA processing [25,26]. Furthermore, *parkin*-mutant fibroblasts showed an altered lipid profile [27], dysfunctions of lysosomal function [28] and of clock gene-dependent energy metabolism [29], and higher Ca^2+^ and cAMP basal levels [4,5,30]. 

The higher cAMP level, observed in *parkin*-mutant fibroblasts, appeared linked to an increased expression of soluble adenylate cyclase (sAC), which produces cAMP, and to a lower expression of the phosphodiesterase isoform 4 (PDE4), which hydrolyzes cAMP and inactivates cAMP-mediated signaling [30]. PDE4 is the major isoform of the phosphodiesterase family, prominently expressed in fibroblasts and brain, and often associated with various pathophysiological conditions [31]. 

The higher basal intracellular Ca^2+^ level in the cytosol and, in particular, in the mitochondria could be responsible for the increased sAC-dependent cAMP level in *parkin*-mutant fibroblasts [30]. cAMP is one of the main second messengers proved to modulate mitochondrial metabolism [32,33,34,35,36,37,38], and it is strictly linked to Ca^2+^ homeostasis [39,40] that, in turn, is also involved in the regulation of mitochondria functions [41]. 

Resveratrol is a well-known nonflavonoid polyphenol endowed with powerful antioxidant properties, primarily found in red grapes/wine [42] and reported to have protective effects in several neurodegenerative diseases [43,44]. Evidences have been provided, in *parkin*-mutant fibroblasts, on the resveratrol ability to induce a partial rescue of mitochondrial dysfunctions [5]. Briefly, the resveratrol treatment induced an energy homeostasis improvement through activation of the AMP-mediated protein kinase (AMPK) pathway, resulting in increased expression of several PGC1α target genes involved in mitochondrial biogenesis and radical oxygen homeostasis [5]. It is reported that resveratrol modulates cellular cAMP level and Ca^2+^ homeostasis by inhibiting PDE4 and plasma membrane Ca^2+^-ATPase (PMCA) in C2C12 myotube and fibroblast cell cultures, respectively. Moreover, resveratrol modulates the expression of proteins present at the contact sites between mitochondria and endoplasmic reticulum (ER) in different cellular systems [45,46,47,48,49,50,51]. 

Organelle functions are strictly associated with the capacity to bind other organelles via membrane contact sites [52]. Specific contact sites are present between mitochondria and endoplasmic reticulum (ERMCSs) where several Ca^2+^-transport systems are localized [53,54,55,56,57,58]. The Ca^2+^ signaling plays a central role in the cellular function regulating autophagy, mitochondrial metabolism, and cell death [41]. Recently, PINK1 and parkin have been found to be mainly localized at the ERMCSs and, together with other several proteins, control the crosstalk between the two organelles [59,60]. ERMCSs represent essential structures, linked to multiple pathways, among which Ca^2+^ homeostasis, lipid transfer, autophagy, and mitochondrial dynamics [61,62,63] whose perturbations are associated with several diseases, including neurological disorders [64]. In mammals, several tethering molecules are involved in the formation of ERMCSs [65], among which are glucose-regulated protein 75 (GRP75), mitochondrial Rho GTPase 2 (Miro2), and mitofusin 2 (Mfn2). GRP75 physically bridges VDAC1, an outer mitochondrial membrane (OMM) protein, to the inositol 1,4,5-trisphosphate receptor (IP3R), an ER Ca^2+^-release channel [63,66]. Miro2 is an OMM protein that anchors mitochondria to microtubules and is required for normal mitochondrial cristae architecture, mitochondrial transport, and mitochondria-associated membranes (MAMs) function [67]. Mfn2 is a large GTPase, located on both the OMM and ER surface, which forms heterocomplexes with Mfn1. Indeed, Mfn2 has been found enriched at ERMCSs, where it regulates organelles tethering in different tissues [68]. Moreover, Miro2 and Mfn2 have been found to take part in the regulation of the Ca^2+^ flux from the ER to mitochondria [69].

In this study we evaluated the effect of resveratrol treatment on the basal cellular cAMP and Ca^2+^ levels and on the expression of specific proteins localized at ERMCSs in control and *parkin-*mutant fibroblasts, lacking the 50 kDa full-length parkin protein. The results attained suggest that resveratrol treatment of *parkin*-mutant fibroblasts induced a remodulation of the cAMP homeostasis by decreasing transmembrane adenylyl cyclases (tmAC) and increasing sAC contribution to cAMP level. In addition, resveratrol affects Ca^2+^ homeostasis, inducing a significant increase of cytosolic and mitochondrial Ca^2+^ levels. These effects might be additionally associated with a restoration of normal levels of Miro2 and Mfn2, upregulated in *parkin*-mutant fibroblasts.

## 2. Materials and Methods

### 2.1. Cell Cultures

*Parkin*-mutant fibroblasts from a patient affected by an early-onset PD, with compound heterozygous mutations (del exon7–9/Glu409X), lacking the 50 kDa full-length parkin protein, and control fibroblasts from one healthy subject, were obtained by explants from skin punch biopsy, after informed consent, and cultured as previously described [5,30]. Control and *parkin*-mutant primary fibroblasts were grown in a T25 Flask and used for experiments at 80% confluence. For resveratrol treatment the cells were incubated with dimethyl sulfoxide (0.02% DMSO), used as control vehicle, or 25 μM resveratrol (RSV) (Sigma Aldrich, St. Louis, MO, USA, Catalog number: R5010) for 30 min at 37 °C. Following extensive dose-dependence assays, a resveratrol concentration of 25 μM was chosen for the absence of cytotoxicity up to 48 h of treatment. 

### 2.2. Cyclic Adenosine Monophosphate (cAMP) Assay

Control and RSV-treated cells were incubated in the absence or in the presence of 10 µM Rolipram, 100 µM 3-Isobutyl-1-methylxanthine (IBMX), 10 µM forskolin, and 100 µM SQ22536 for 30 min at 37 °C. For cAMP assays, the culture medium was removed and 1 mL of 0.1 M HCl was added to the cell layer, followed by 10 min incubation at 37 °C. The lysed cells were scraped, transferred into tubes, and centrifuged at 1300× *g* for 10 min at 4 °C. The supernatant was used for cAMP measurements using the direct cAMP ELISA Kit (Enzo Life Sciences, New York, NY, USA) according to the manufacturer’s instruction. Measurements were performed on a Victor 2030 multilabel reader (PerkinElmer, Waltham, MA, USA). The cAMP values were normalized to the protein concentration and expressed as pmol/mg protein.

### 2.3. Quantitative Fluorimetric Measurement of Cytosolic and Mitochondrial Ca^2+^ Levels

Cytosolic and mitochondrial Ca^2+^ levels were measured in control and *parkin*-mutant fibroblasts exposed to either vehicle (0.02% DMSO) or 25 μM resveratrol (RSV) for 30 min, by using the cell-permeable fluorescent Ca^2+^ indicator Fluo-4 AM or X-Rhod-1AM (Invitrogen, Carlsbad, CA, USA), respectively [70,71]. Once inside the cell, the lipophilic blocking groups of Fluo-4 AM are cleaved by nonspecific cell esterase, resulting in a negatively charged dye that stays inside cells, and whose fluorescence is greatly enhanced upon binding to Ca^2+^. X-Rhod-1AM is a cell permeable cationic fluorescent dye which can result in a membrane potential-driven uptake into mitochondria. For the Ca^2+^ level measurements, the cells at 80% confluence were incubated with 2.5 μM of the fluorescent probes for 30 min at 37 °C. Cell monolayers collected by trypsinization and centrifugation were resuspended in a buffer containing 10 mM Hepes and 6 mM D-Glucose (pH 7.4) at an approximate concentration of 1 × 10^5^ cells in 1 mL. Fluorescence intensity was measured at 25 °C in a spectrofluorometer (Jasco FP6200 Mary’s Court Easton, MD, USA), equipped with a stirrer and temperature control, by the subsequent addition of 5 mM CaCl_2_, 0.1% Triton X-100 (for cytosolic Ca^2+^ level detection), 0.1% Na-Colate (for mitochondrial Ca^2+^ level detection), and 40 mM EGTA. The excitation/emission wavelengths were 495 nm/506 nm for Fluo-4 AM and 580 nm/602 nm for X-Rhod-1 AM. The cytosolic and mitochondrial Ca^2+^ levels were evaluated by using an apparent Kd (443 nM for Fluo-4AM and 700 nM for X-Rhod-1AM) according to the equation described by Grynkiewicz [72]. Where indicated, incubations with DMSO or RSV, in the presence or in the absence of 1 µM thapsigargin (TG), 10 µM dantrolene (Dan), 5 µM ruthenium red (RR), were performed for 30 min at 37 °C.

### 2.4. Western Blot Analysis

Total cell proteins (20 μg) from control and *parkin*-mutant fibroblasts exposed to either vehicle (0.02% DMSO) or 25 μM resveratrol (RSV) for 24 h were separated on a 8% Tris-Glycine SDS–PAGE, transferred to nitrocellulose membranes with 0.2 µm pore size (Bio-Rad, Hercules, CA, USA), and immunoblotted with specified primary antibodies against GRP75 (1:200; Santa Cruz Bio Technology, Dallas, TX, USA, Catalog number: sc-13967), Miro2 (1:1000; Cell Signaling Technology, Danvers, MA, USA, Catalog number: #14016), and Mfn2 (1:200; Millipore, Burlington, MA, USA, Catalog number: #ABC42). Sample loading was assessed with actin (1:3000; Sigma Aldrich, St. Louis, MO, USA, Catalog number: A1978). After incubation with the horseradish peroxidase-conjugated secondary antibody (1:3000; Bio-Rad, Hercules, CA, USA, Anti-mouse catalog number: #1706516; Anti-rabbit catalog number: #1707515), signals were settled by the chemiluminescence kit (ClarityTM Western ECL Substrate, Bio-Rad, Hercules, CA, USA). Immuno-revealed bands were acquired by ChemiDoc Imaging System XRS (Bio-Rad, Hercules, CA, USA) and analyzed with the Image J Lab software 1.8.0_112 (https://imagej.nih.gov/ij/index.html accessed on 21 July 2021). 

### 2.5. Protein Measurement

The protein concentration was measured by the Quick Start™ Bradford Protein Assay (Bio-Rad, Hercules, CA, USA) and bovine serum albumin was used as the standard.

### 2.6. Statistical Analysis

Data are shown as mean ± SEM. The significance of any differences throughout the data sets presented was determined by one-way or two-way analysis of variance (ANOVA) with the Tukey post hoc test. The threshold for statistical significance (*p*-value) was set to 0.05.

## 3. Results

### 3.1. Resveratrol Decreases cAMP Level in Parkin-Mutant Fibroblasts

**Figure 1:**
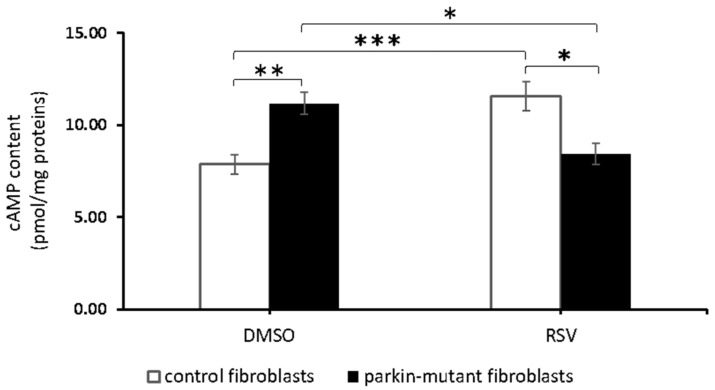
Effect of resveratrol on the basal cAMP cellular level in control and *parkin*-mutant fibroblasts. Basal cyclic adenosine monophosphate (cAMP) cellular level, expressed as pmol/mg protein, in control (open bar) and *parkin*-mutant (black bar) fibroblasts exposed to either vehicle (DMSO) or 25 μM resveratrol (RSV) for 30 min. The values represent the means ± SEM from 3 independent experiments under each condition. The significance was determined by two-way ANOVA with Tukey post hoc; *, *p* ˂ 0.01; **, *p* ˂ 0.001; ***, *p* ˂ 0.0005. For more details, see Materials and Methods.

We previously showed a higher basal level of cAMP in *parkin*-mutant fibroblasts compared to control cells [30]. To examine the effect of the resveratrol (RSV) on the basal level of cAMP, we treated control and *parkin*-mutant fibroblasts with 25 μM RSV or vehicle for 30 min, as described in Materials and Methods. RSV-treatment induced a significant increase of cAMP level in control fibroblasts and, on the contrary, a significant decrease of the higher basal cAMP level present in *parkin*-mutant fibroblasts (Figure 1). To note, the cAMP level in RSV-treated *parkin*-mutant fibroblasts reached a value comparable to that of control fibroblasts under basal conditions. 

**Figure 2:**
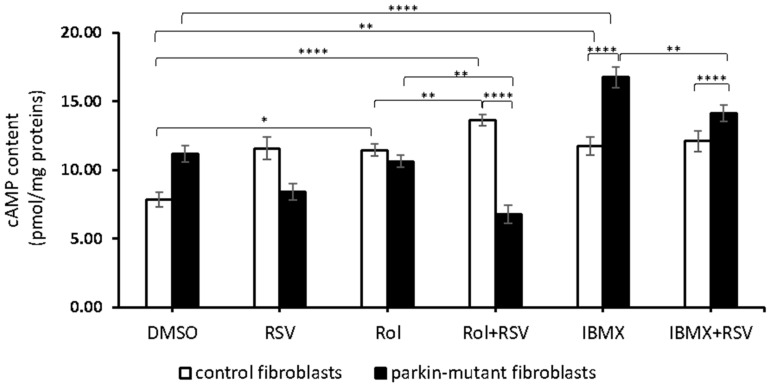
Effect of resveratrol on the cAMP cellular level in the presence of rolipram and IBMX. cAMP content in control (open bar) and *parkin*-mutant (black bar) fibroblasts exposed for 30 min to vehicle (DMSO) or 25 μM resveratrol (RSV). Where indicated, the cells were treated for 30 min with 10 µM rolipram (Rol) or 100 µM IBMX (IBMX) alone or co-incubated with resveratrol, Rol+RSV, or IBMX+RSV. The values represent the means ± SEM from 3 independent experiments under each condition. The significance was determined by two-way ANOVA with Tukey post hoc; *, *p* ˂ 0.01; **, *p* ˂ 0.001; ****, *p* ˂ 0.0001. The statistical significance of cAMP level among DMSO and RSV treatments is presented in Figure 1 and omitted herein to streamline the figure. For more details, see Materials and Methods.

Since RSV is reported to modulate cAMP level by inhibiting PDE4 [45], we carried out experiments in the presence of rolipram (Rol), a selective inhibitor of PDE4. Rol-treatment led to a significant increase of the cAMP level in control fibroblasts without any significant effect in *parkin*-mutant fibroblasts, thus suggesting a lower expression of PDE4 [30]. The co-treatment with resveratrol (Rol+RSV) induced a further increase of cAMP level in control cells and a decrease in *parkin*-mutant fibroblasts as compared with Rol-treatment (Figure 2). In control cells, the treatment with IBMX, a pan-inhibitor of other phosphodiesterases (PDEs), resulted in a significant increase of the cAMP level [30], comparable with that attained by Rol-treatment, and no further changes were observed by co-treatment with resveratrol (Figure 2). In *parkin*-mutant fibroblasts, IBMX-treatment caused a significant increase of the cAMP level, much higher than in control cells [30], which was, however, significantly reduced in the presence of RSV (Figure 2). These results suggested that RSV, while in control fibroblasts, increased cAMP level, likely inhibiting PDE4 [45] in PDE4-defective *parkin*-mutant cells, and could act on different targets.

As the steady-state level of cAMP results from the balance between its synthesis and degradation, we considered the possibility of an inhibitory effect of RSV on the adenylate cyclases in *parkin*-mutant fibroblasts. 

**Figure 3:**
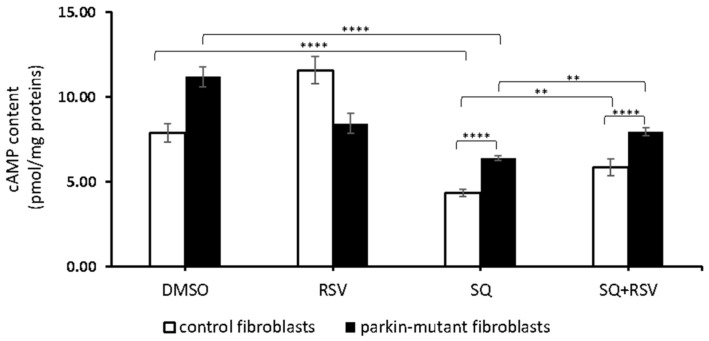
Effect of resveratrol on cAMP cellular level in the presence of SQ22386. cAMP content in control (open bar) and *parkin*-mutant (black bar) fibroblasts exposed for 30 min to vehicle (DMSO) or 25 μM resveratrol (RSV). Where indicated, the cells were incubated for 30 min with 100 µM SQ22386 (SQ) or co-incubated with resveratrol (SQ+RSV). The values represent the means ± SEM from 3 independent experiments under each condition. The significance was determined by two-way ANOVA with Tukey post hoc; **, *p* ˂ 0.001; ****, *p* ˂ 0.0001. The statistical significance of cAMP level among DMSO and RSV treatments is presented in Figure 1 and omitted herein to streamline the figure. For more details, see Materials and Methods.

cAMP can be produced by transmembrane adenylyl cyclases (tmACs) and by soluble adenylyl cyclase (sAC). Therefore, we analyzed the effect of RSV on the cAMP level in the presence of SQ22536 (SQ), a specific inhibitor of tmACs [73]. As already reported [30], the SQ treatment resulted in a significant decrease of the cAMP level, both in control and *parkin*-mutant cells, though its level in *parkin*-mutant cells remained much higher than in control cells. The co-treatment with RSV (SQ+RSV) induced a significant increase of cAMP level with respect to SQ-treatment in both control and *parkin*-mutant fibroblasts (Figure 3). It is noteworthy that, even in the case of co-treatment (SQ+RSV), the level of cAMP in *parkin*-mutant fibroblasts was higher than that observed in control cells in the same experimental condition.

**Figure 4:**
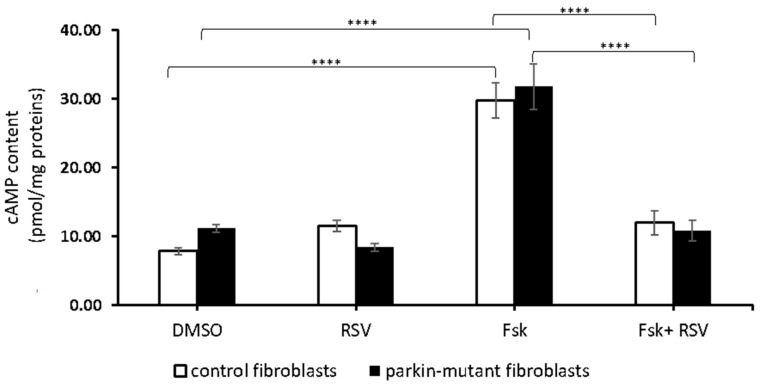
Effect of resveratrol on cAMP cellular level in the presence of forskolin. cAMP content in control (open bar) and *parkin*-mutant (black bar) fibroblasts exposed for 30 min to vehicle (DMSO) or 25 μM resveratrol (RSV). Where indicated, the cells were incubated 30 min with 10µM forskolin (Fsk) or co-incubated with resveratrol (Fsk+RSV). The values represent the means ± SEM from 3 independent experiments under each condition. The significance was determined by two-way ANOVA with Tukey post hoc; ****, *p* ˂ 0.0001. The statistical significance of cAMP level among DMSO and RSV treatments is presented in Figure 1 and omitted herein to streamline the figure. For more details, see Materials and Methods.

Next, we investigated the effect of resveratrol in the presence of forskolin (Fsk), an activator of tmAC. Fsk-treatment resulted in a strong enzymatic response in both control and *parkin*-mutant fibroblasts with a significant increase of cAMP level [28] that was completely prevented by co-treatment with resveratrol (Fsk+RSV) (Figure 4). This result suggested a possible inhibition of tmAC by resveratrol as already described [74].

The reported results need to be reconciled with the opposite effect of resveratrol on the cAMP level observed in control and *parkin*-mutant fibroblasts. A reliable explanation could be that, in RSV-treated control cells, the likely inhibition of tmAC might be compensated by the inhibition of PDE4, thus resulting in an increased cAMP level. The same hypothesis does not apply to the case of RSV-dependent decrease of cAMP level observed in *parkin*-mutant fibroblasts as these cells lack efficient PDE4 activity. 

### 3.2. Resveratrol Further Increases Cytosolic and Mitochondrial Ca^2+^ Levels in Parkin-Mutant Fibroblasts

**Figure 5:**
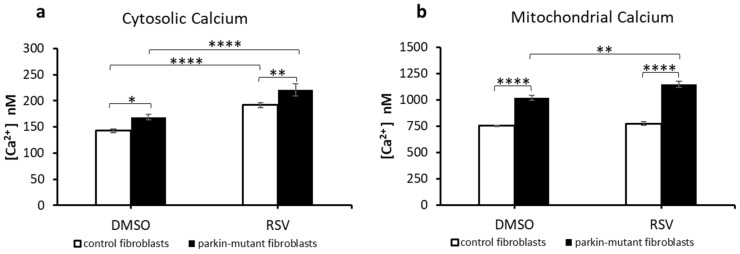
Effect of resveratrol on the basal cytosolic and mitochondrial Ca^2+^ levels in control and *parkin*-mutant fibroblasts. Spectrofluorometric measurements of cytosolic (**a**) and mitochondrial Ca^2+^ (**b**) levels in control (open bar) and *parkin*-mutant (black bar) fibroblasts loaded, respectively, with Fluo-4AM and X-Rhod-1AM, exposed to either vehicle (DMSO) or 25 μM resveratrol for 30 min. The values, expressed as nM, represent the means ± SEM from 3 independent experiments under each condition. The significance was determined by two-way ANOVA with Tukey post hoc; *, *p* ˂ 0.01; **, *p* ˂ 0.001; ****, *p* ˂ 0.0001. For more details, see Materials and Methods.

As previously reported, *parkin*-mutant fibroblasts showed deregulation of basal level of cAMP associated with higher steady state Ca^2+^ basal level in both cytosolic and mitochondrial compartment [28]. Since cAMP is strictly connected to calcium level [75] and, as we previously showed, a calcium overload in fibroblast cell cultures leads to an increase of cAMP [30], we measured the Ca^2+^ level in RSV-treated cells. In agreement with previous studies [47,48,76], RSV-treatment caused a significant increase of cytosolic Ca^2+^ level in both control and *parkin*-mutant cells (Figure 5a). Instead, RSV-treatment caused an increase of mitochondrial Ca^2+^ level only in *parkin*-mutant cells (Figure 5b). Of note, in the RSV-treated *parkin*-mutant fibroblasts, the Ca^2+^ level was significantly higher in both cytosolic and mitochondrial compartments than in control fibroblasts.

**Figure 6:**
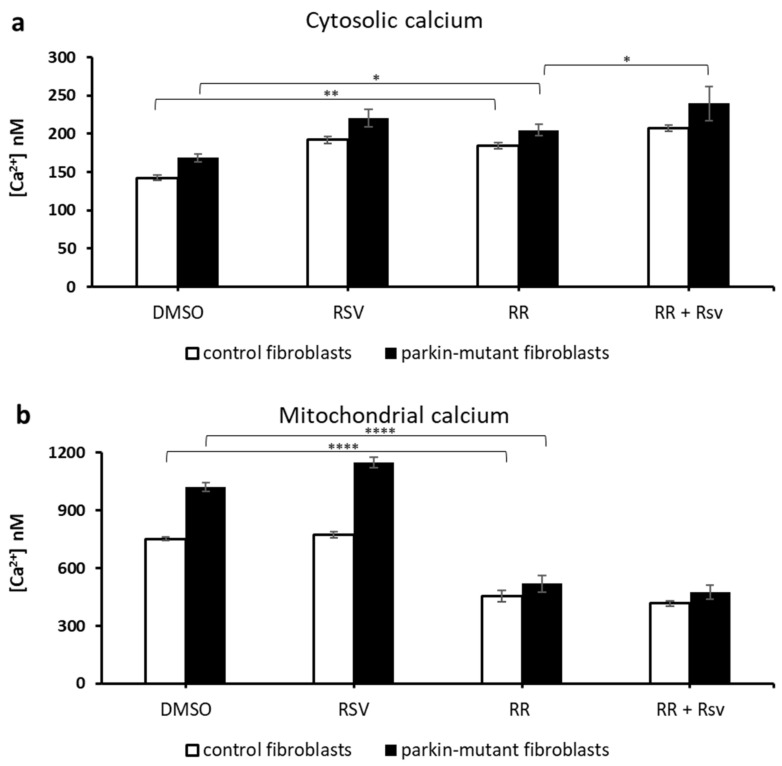
Effect of resveratrol on the basal cellular Ca^2+^ level in control and *parkin*-mutant fibroblasts in the presence of ruthenium red (RR). Spectrofluorometric measurements of cytosolic (**a**) and mitochondrial (**b**) Ca^2+^ levels in control (open bar), and *parkin*-mutant (black bar) fibroblasts loaded, respectively, with Fluo-4AM and X-Rhod-1AM. The cells were exposed for 30 min to vehicle (DMSO) or 25 μM resveratrol (RSV). Where indicated, the cells were incubated for 30 min with 5 µM ruthenium red (RR) alone or co-incubated with resveratrol (RSV+RR). The values, expressed as nM, represent the means ± SEM from 3 independent experiments under each condition. The significance was determined by two-way ANOVA with Tukey post hoc; *, *p* ˂ 0.01; **, *p* ˂ 0.001; ****, *p* ˂ 0.0001. The statistical significance of Ca^2+^ level among DMSO and RSV treatments is presented in Figure 5 and omitted herein to streamline the figure. For more details, see Materials and Methods.

Treatment with ruthenium red (RR), a specific inhibitor of the mitochondrial Ca^2+^ uniporter (MCU) [77,78,79,80], induced a significant increase of cytosolic and a decrease of mitochondrial steady-state Ca^2+^ levels, both in control and *parkin*-mutant fibroblasts [30]. Co-treatment with resveratrol (RR+RSV) caused a further increase of cytosolic Ca^2+^ in *parkin*-mutant fibroblasts, as compared with RR-treated cells (Figure 6a), but no additional effect on the mitochondrial Ca^2+^ level (Figure 6b) was observed. 

The cytosolic and the mitochondrial levels of Ca^2+^ are linked to ion release/uptake fluxes mainly controlled by intracellular stores. Taking in the account that parkin, localized at the ERMCSs, modulates at least in vitro ER mitochondria Ca^2+^ crosstalk [81,82,83,84], we tested the effect of thapsigargin (TG), a specific irreversible inhibitor of the ER Ca^2+^-ATPase (SERCA) [85] and of dantrolene (Dan), an inhibitor of the ryanodine receptor (RyR), an ER-resident Ca^2+^ -release channel [86].

**Figure 7:**
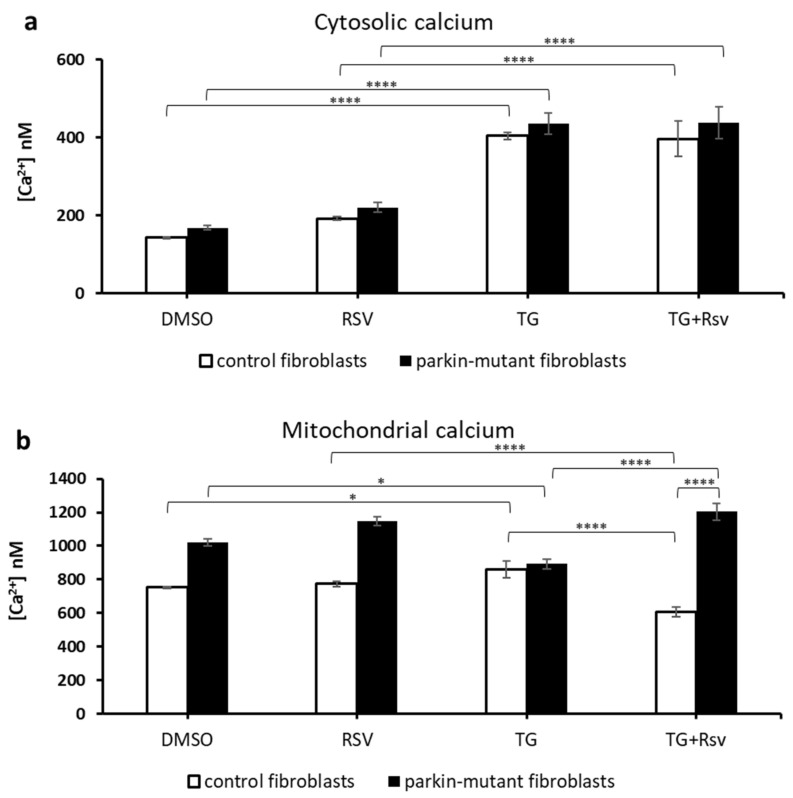
Effect of resveratrol on the basal cellular Ca^2+^ level in control and *parkin*-mutant fibroblasts in the presence of thapsigargin (TG). Spectrofluorometric measurements of cytosolic (**a**) and mitochondrial (**b**) Ca^2+^ levels in control (open bar), and *parkin*-mutant (black bar) fibroblasts loaded, respectively, with Fluo-4AM and X-Rhod-1AM. The cells were exposed for 30 min to vehicle (DMSO) or 25 μM resveratrol (RSV). Where indicated, the cells were incubated for 30 min with 1 µM thapsigargin (TG) alone or co-incubated with resveratrol (RSV+TG). The values, expressed as nM, represent the means ± SEM from 3 independent experiments under each condition. The significance was determined by two-way ANOVA with Tukey post hoc; *, *p* ˂ 0.01; ****, *p* ˂ 0.0001. The statistical significance of Ca^2+^ level among DMSO and RSV treatments is presented in Figure 5 and omitted herein to streamline the figure. For more details, see Materials and Methods.

TG-treatment resulted in a significant increase of the cytosolic Ca^2+^ level in both control and *parkin*-mutant fibroblasts [30]. A small but significant increase of mitochondrial Ca^2+^ was observed in control fibroblasts, while a decrease of it was observed in *parkin*-mutant fibroblasts [30]. Co-treatment of TG with RSV (TG+RSV) did not cause appreciable changes in the cytosolic Ca^2+^ level in both cell samples as compared with that elicited by TG alone (Figure 7a), remaining significantly higher with respect to RSV-treatments. Conversely, the co-treatment (TG+RSV) caused a significant decrease of the mitochondrial Ca^2+^ level in control cells with respect to both TG-treatment and RSV-treatment and an increase in *parkin*-mutant fibroblasts compared to TG-treated cells (Figure 7b). Thus, although in control and *parkin*-mutant fibroblasts the TG treatment induced the same effect on Ca^2+^ level in both mitochondrial and cytosolic compartment, the co-treatment with RSV showed an opposite effect in mitochondria, inducing a decrease of Ca^2+^ level in control cells and an increase in *parkin*-mutant fibroblasts.

**Figure 8:**
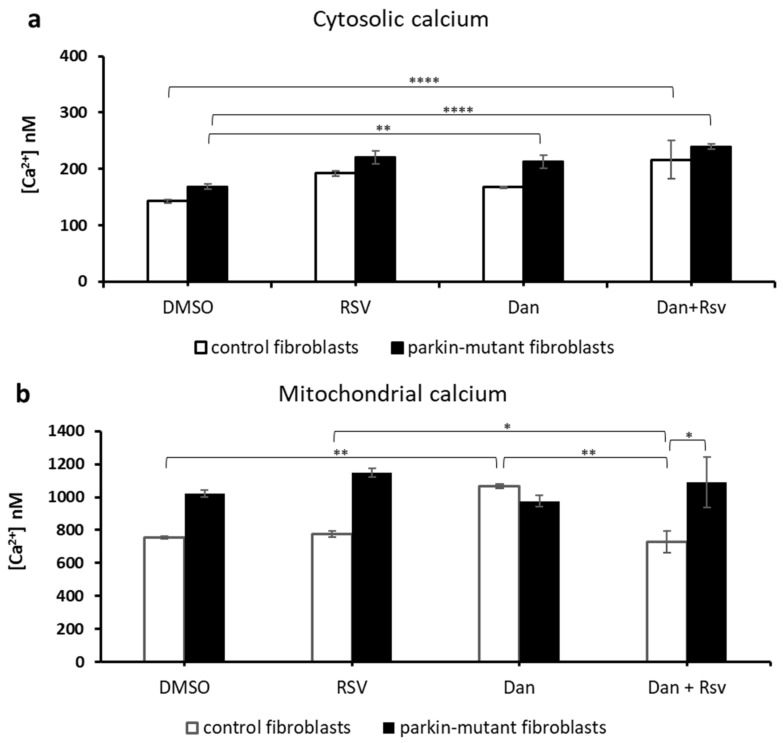
Effect of resveratrol on the basal cellular Ca^2+^ level in control and *parkin*-mutant fibroblasts in the presence of dantrolene (Dan). Spectrofluorometric measurements of cytosolic (**a**) and mitochondrial (**b**) Ca^2+^ levels in control (open bar), and *parkin*-mutant (black bar) fibroblasts loaded, respectively, with Fluo-4AM and X-Rhod-1AM. The cells were exposed for 30 min to vehicle (DMSO) or 25 μM resveratrol (RSV). Where indicated, the cells were incubated for 30 min with 10 µM dantrolene (Dan) alone or co-incubated with resveratrol (RSV+Dan). The values, expressed as nM, represent the means ± SEM from 3 independent experiments under each condition. The significance was determined by two-way ANOVA with Tukey post hoc; *, *p* ˂ 0.01; **, *p* ˂ 0.001; ****, *p* ˂ 0.0001. The statistical significance of Ca^2+^ level among DMSO and RSV treatments is presented in Figure 5 and omitted herein to streamline the figure. For more details, see Materials and Methods.

Regarding the effect elicited by Dan, we observed a slight but significant increase of cytosolic Ca^2+^ in *parkin*-mutant fibroblasts [30]. To note, Dan-treatment caused a significant increase of mitochondrial Ca^2+^ level in control cells and the co-treatment (Dan+RSV) induced a decrease of calcium level, which was lower than that observed with RSV alone (Figure 8a). No significant effect on mitochondrial Ca^2+^ level was observed in *parkin*-mutant fibroblasts irrespective of whether dantrolene was tested alone or in combination with RSV (Figure 8b). It is noteworthy that, even in the case of co-treatment (Dan+RSV), the level of mitochondrial Ca^2+^ in *parkin*-mutant fibroblasts was significantly higher than that observed in control cells (Figure 8b). On this basis we hypothesize that RSV-mediated enhancement of the Ca^2+^ level could be due to an altered Ca^2+^ exchange between ER and mitochondria. 

### 3.3. Parkin-Mutant Fibroblasts Show Higher Levels of GRP75, Miro2, and Mfn2 Proteins; Resveratrol Treatment Decreases Miro2 and Mfn2 Protein Levels 

Several soluble and integral membrane proteins provide both structural and functional features in keeping the distance between mitochondria and ER in a proper range and in controlling inter-organelle Ca^2+^ homeostasis [62,87,88,89]. Therefore, we investigated the expression of some proteins known to be involved in the tethering/modulation of mitochondria-ER interface: GRP75, Miro2, and Mfn2. 

**Figure 9:**
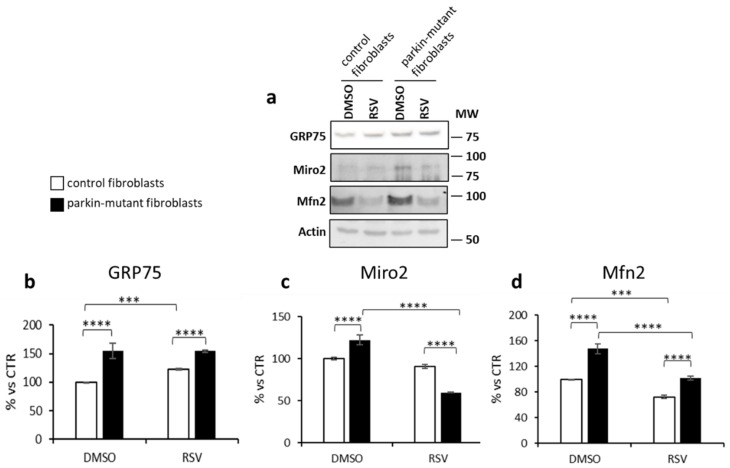
Effect of resveratrol on protein levels of GRP75, Miro2, and Mfn2 in control and *parkin*-mutant fibroblasts. (**a**) Representative image of Western blot of GRP75, Miro2, and Mfn2 performed on whole cell lysates from control and *parkin*-mutant fibroblasts exposed to either vehicle (DMSO) or 25 μM resveratrol for 24 h (MW, molecular weight). The graphs (panel (**b**), GRP75; panel (**c**), Miro2; panel (**d**), Mfn2) display the statistical densitometric analysis of band intensity of proteins normalized to the corresponding actin level, used as loading control. Data means ± SEM from 3 independent experiments under each condition are expressed as percentage of vehicle-treated control cells. The significance was determined by one-way ANOVA with Tukey post hoc; ***, *p* ˂ 0.0005; ****, *p* ˂ 0.0001. For more details, see Materials and Methods.

Western blotting and densitometric analysis showed higher basal level of GRP75, Miro2, and Mfn2 in *parkin*-mutant fibroblasts, as compared to control cells (Figure 9). Interestingly, 24 h of RSV-treatment appeared to affect the level of these ER–mitochondria tethering proteins. In particular, GRP75 was significantly upregulated in control cells whereas Miro2 and Mfn2 were downregulated to a larger extent in *parkin*-mutant cells than in control cells.

## 4. Discussion

Resveratrol is a natural polyphenolic compound with antioxidant and anti-inflammatory properties, able to modulate many cellular processes, including mitochondrial activity and ion homeostasis. These properties are not simply linked to the direct ROS scavenging activity of resveratrol but also to its ability to bind and modulate several intracellular targets [90,91,92]. Resveratrol can increase cytosolic Ca^2+^ in many cell types [46,47,48,76] by modulation of specific pathways involved in Ca^2+^ homeostasis. In our previous studies, we examined the effect of resveratrol treatment on *parkin*-mutant fibroblasts [5]. We showed that resveratrol induced an increase of mitochondrial complex I activity with a consequent significant increase of mitochondrial ATP content and a decrease in lactate level, suggesting a switch from glycolytic to oxidative metabolism. The resveratrol-dependent improvement of the mitochondrial oxidative function has been associated with a reduced oxidative stress and an increased expression of several PGC1α target genes involved in mitochondrial biogenesis. These effects have been linked to the AMPK-dependent SIRT1 activation [5] (see also [93,94,95,96,97]). In addition, in the same cellular model of *parkin-*mutant fibroblasts, we observed an altered mitochondrial cAMP and Ca^2+^ homeostasis [30]. It has been described that resveratrol can activate the CaMKKβ-AMPK pathway controlling both Ca^2+^ and cAMP homeostasis [45,98,99].

In this study, we assessed the effect of resveratrol-treatment on the altered mitochondrial Ca^2+^ and cAMP homeostasis in a cellular model of *parkin*-mutant fibroblasts. We first observed that resveratrol-treatment induced an increase of cAMP level in control cells, likely due to inhibition of PDE4 [45], and a decrease in *parkin*-mutant fibroblasts where the PDE4 is less expressed [30]. Taking into account that the cellular cAMP basal level in the *parkin*-mutant fibroblasts is higher than in control cells, we want to highlight that in resveratrol-treated *parkin*-mutant cells, the cAMP level decreased to a value comparable to the basal level observed in control cells. In addition, resveratrol-treatment induced a large decrease of the forskolin-stimulated adenylate cyclase activity in both control and *parkin*-mutant fibroblasts, thereby indicating a likely inhibitory effect of resveratrol on the tmACs. Thus, in *parkin*-mutant cells the inhibition of tmAC by resveratrol, not sufficiently compensated by an efficient PDE4 activity, could be responsible for the observed decrease in the cAMP level. These results are in agreement with the significant inhibition of forskolin-stimulated tmAC activity by low concentrations of resveratrol mediated by binding to AdoRs, observed in a glial cell model [74]. Further observations on the role of resveratrol in the modulation of cAMP level stemmed from experiments in the presence of SQ, a tmAC inhibitor. Taking into account that resveratrol inhibits PDE4 [45], it is conceivable that, in resveratrol-treated control cells, the observed increase of cAMP level, in spite of tmAC inhibition (in the presence of SQ), should be due to PDE4 inhibition. Conversely, in *parkin*-mutant cells, lacking the PDE4, the increase in cAMP level, observed in these conditions, should be linked to the resveratrol-dependent increase of mitochondrial Ca^2+^ which, in turn, primed sAC activity. 

Since resveratrol activates AMPK/SIRT1/PGC1α signaling in control and *parkin*-mutant fibroblasts [5], and considering that Ca^2+^ modulates the sAC-dependent cAMP level and the Ca^2+^/CaMKKβ pathway activating the AMPK [98,99], we pointed the attention to the modulation of Ca^2+^ level by resveratrol. Higher basal Ca^2+^ level both in the cytosolic and, mainly, in the mitochondrial compartment has been already shown in *parkin*-mutant fibroblasts than in control cells [30]. Of note, proteomics studies in *parkin*-mutant fibroblasts showed downregulation of several Ca^2+^-binding proteins [26] among which calreticulin, a chaperone protein engaged in ER Ca^2+^ storage capacity [100], and three proteins of the S100 family, S100-A4, S100-A6, and S100-A10, involved in Ca^2+^-dependent regulation of a variety of intracellular activities such as intracellular Ca^2+^ homeostasis [101]. Resveratrol-treatment induced a further increase of cytosolic Ca^2+^ level in both control and *parkin*-mutant fibroblasts and an increase of the mitochondrial Ca^2+^ in *parkin*-mutant cells (see also [102]). The release of Ca^2+^ from extracellular and intracellular compartment, induced by resveratrol-treatment, could be responsible for the AMPK-dependent restore of mitochondrial respiration and ATP production previously described in *parkin*-mutant fibroblasts [5]. 

As the cellular Ca^2+^ homeostasis depends on various Ca^2+^ channels and active pumps, including MCU, SERCA, and RyR, which control Ca^2+^ release and uptake from intracellular stores, we evaluated on these the effect of resveratrol using specific inhibitors. Mitochondrial Ca^2+^ uptake is largely mediated by the MCU and driven by the mitochondrial membrane potential [103]. Parkin selectively regulates the turnover of MICU1, a subunit of MCU [104], by promoting its proteasome-mediated degradation. The loss of function in the *parkin*-mutant fibroblasts should enhance the MCU-mediated entry of Ca^2+^ into the mitochondria [see [30]]. The results obtained by the co-treatment with resveratrol and ruthenium red (RR), in *parkin*-mutant fibroblasts, showed a further increase in cytosolic Ca^2+^ level as compared with RR-treated cells and the absence of any effect on the mitochondrial Ca^2+^ level, therefore suggesting that resveratrol is not acting on MCU. 

The endoplasmic reticular Ca^2+^ ATPase (SERCA) is involved in maintaining the low resting Ca^2+^ concentration in cytosolic compartment. In both control and *parkin*-mutant fibroblasts, the co-treatment with resveratrol and thapsigargin (TG+RSV) did not further increase the high cytosolic level of Ca^2+^ elicited by TG alone. This leads to assume a limited inhibition of SERCA by resveratrol as already described [105]. Furthermore, in the TG+RSV-treated control cells, we observed a larger decrease in mitochondrial Ca^2+^ level as compared to the TG-treated cells. This result is in agreement with the role of mitochondria in providing a local source of Ca^2+^ for ER refilling in Ca^2+^-depleted ER [106]. On the contrary, in *parkin*-mutant fibroblasts, the co-treatment TG+RSV induced an increase of mitochondrial Ca^2+^ level, as compared with TG-treated cells, showing a level of Ca^2+^ comparable to that measured in the presence of resveratrol alone. In *parkin*-mutant cells the TG+RSV co-treatment seems to prevent or, in any case, not to allow the mitochondrial Ca^2+^ ER refilling in Ca^2+^-depleted ER cells. 

Previous studies clearly established in MAMs a functional and structural communication between mitochondria and ER [107,108,109,110], characterized by the presence of ryanodine receptors (RyRs) and inositol 1,4,5-triphosphate receptors (Ins (1,4,5) P3Rs). ER and the nearby mitochondria create microdomains through the VDACs and the MCU complex where intracellular Ca^2+^ transfer from ER to mitochondria takes place [62]. Co-incubation of resveratrol with dantrolene (Dan+RSV) induced a larger increase of Ca^2+^ level in the cytosolic compartment, in both control and *parkin*-mutant fibroblasts but a marked decrease of the mitochondrial Ca^2+^ only in control cells, as compared with Dan-treated cells. Under these conditions, in control cells, resveratrol could induce a partial inhibition of SERCA, causing an increase in cytosolic Ca^2+^ level and, as described for the TG-treatment, this could induce the ER refilling by the mitochondria. In *parkin*-mutant fibroblasts this process, even in this case, does not appear to occur and upon co-treatment (Dan+RSV) no significant changes of mitochondrial Ca^2+^ level were observed, as compared with Dan-treated cells. 

In *parkin*-mutant fibroblasts, the increased resveratrol-dependent Ca^2+^ level could be responsible for the enhanced activity of intramitochondrial Ca^2+^-sensitive dehydrogenases. This leads to an increased supply of reducing equivalents for the respiratory chain activity and consequent increase of ATP synthesis [5,111]. We assumed that, in *parkin-*mutant fibroblasts, the basal high level of mitochondrial Ca^2+^ is related to dysfunctional mitochondria, mainly derived by the failed auto(mito)phagic process. In this context, resveratrol-induced Ca^2+^ increase could lead to an improvement of the oxidative phosphorylation system and oxidative stress condition in new functional mitochondria derived from a rebalanced mitochondrial biogenesis vs. mitophagy, resulting from the Ca^2+^-dependent AMPK/SIRT1/PGC1α activation. 

The deregulation of Ca^2+^ homeostasis, in *parkin*-mutant cells, is object of debate. It is reported that *parkin*-null cells and fibroblasts expressing mutant parkin showed reduced ER–mitochondria contact sites associated with a decrease in mitochondrial Ca^2+^ [82]. Conversely, it has been found that the number of ER–mitochondria contact sites is augmented in fibroblasts from PARK2 knockout mice and in human fibroblasts harboring PARK2 mutations [84]. In addition, PINK1 deficiency results in mitochondrial Ca^2+^ overload associated with a lower threshold of mPTP-opening, making neurons vulnerable to apoptosis [112]. Although it is known that Ca^2+^ stimulates the mitochondrial respiratory chain, an excessive Ca^2+^ load is dangerous for mitochondria by opening mPTP, which results in mitochondrial membrane potential dissipation and respiratory chain uncoupling, associated with a decrease of mitochondrial ATP synthesis [113], culminating in cell death. Conversely, a decrease of mitochondrial Ca^2+^ uptake causes a reduction of mPTP opening making the cells resistant to apoptosis (for review see [41]). However, although in *parkin-*mutant fibroblasts we observed a further increase of mitochondrial Ca^2+^ after resveratrol treatment along with an increase of mitochondrial respiration and mitochondrial ATP production [5], we are inclined to rule out any involvement of the mPTP. In addition, it has been shown that resveratrol inhibited the mPTP opening [114,115]. Thus, in *parkin*-mutant fibroblasts, characterized by a deregulation of the crosstalk between cAMP and Ca^2+^, together with ER stress [25], resveratrol treatment can normalize the cAMP content and modulate Ca^2+^ level. This elucidates the mechanism by which resveratrol, by modulating both cAMP and Ca^2+^ levels, restored OXPHOS efficiency through AMPK/SIRT1/PGC1 α activation [5].

In keeping the importance of preserving a proper Ca^2+^ transfer between ER and mitochondria, we studied the effect of 24 h resveratrol-treatment on the expression level of GRP75, Mfn2, and Miro2, three proteins involved in MAMs. Noteworthy, we observed a higher expression level of GRP75 in *parkin*-mutant fibroblasts, as compared with control cells. GRP75 is part of a multiprotein complex gathering IP3R and VDAC1, functionally coupling ER and mitochondria and promoting Ca^2+^ exchanges [63]. In mouse primary neurons, it has been reported that the GRP75 overexpression induces an increase of ER–mitochondria tethering and of mitochondrial Ca^2+^ level [116,117]. Therefore, the high GRP75 protein level observed in *parkin*-mutant fibroblasts could contribute to the higher basal Ca^2+^ level measured in the mitochondrial compartment. 

Moreover, *parkin*-mutant fibroblasts showed a higher expression of Mfn2 as compared with control cells, which was decreased by resveratrol-treatment. Mfn2 is mainly localized at the MAM-related contact sites [118,119,120,121], though its specific function is still matter of debate [68,122]. It has been shown that, in several cell lines, parkin selectively ubiquitinates mammalian Mfn1 and Mfn2 [123,124] for degradation. This is consistent with the higher basal protein level observed in *parkin*-mutant fibroblasts. In primary fibroblasts from *parkin* knockout mice and from *parkin*-mutant fibroblasts, a recent study showed an augmented ER–mitochondria tethering and ER-to-mitochondria Ca^2+^ transfer, likely due to increased Mfn2 level in MAMs [84]. Furthermore, it is also reported that Mfn2 suppression is associated with an increased number of ER–mitochondria contact sites and an increased Ca^2+^ transfer between the two organelles [119]. In the present study, in *parkin*-mutant fibroblasts, the high expression level of Mfn2, likely due to the lack of its ubiquitination, could be involved in the increased steady-state Ca^2+^ level. 

Rho GTPases Miro1/2, localized in the mitochondrial outer membrane, are components of a complex that anchors mitochondria to motor proteins. Their ubiquitination by parkin leads to mitochondrial arrest that further facilitates the elimination of impaired mitochondria by mitophagy [125,126]. Recent studies revealed the role of Miro, containing two Ca^2+^-sensing EF hand domains, in the ER–mitochondria contact sites regulation [67,69]. Furthermore, the PINK1–parkin pathway should negatively regulate Miro level, through ubiquitination, resulting in an increased Miro protein level in PINK1 mutant mammalian cells [125]. Consistently, knock-down of Miro by RNAi decreased mitochondrial Ca^2+^ level in PINK1 mutant dopaminergic neurons [127]. As previously reported for GRP75 and Mfn2, we observed a higher expression level of Miro2 in *parkin*-mutant fibroblasts, which could be responsible of the high Ca^2+^ level therein. Furthermore, it is worth mentioning that the ER stress upregulates GRP75 [128] and Mfn2 [129] expressions, leading to increased MAM formation and mitochondrial Ca^2+^ overload, exactly as observed by our group in *parkin*-mutant cells. 

Parkin plays a central role in the mitochondrial quality-control processes [13] in which a fine balance of mitochondrial autophagy and biogenesis is established [130]. We previously showed that resveratrol treatment caused an enhanced macroautophagic flux through an LC3-independent pathway activation [5]. This effect could be linked to the resveratrol-induced decrease of Miro2 and Mfn2 levels observed in *parkin*-mutant fibroblasts. Thus, the resveratrol treatment, by modulating specific signaling pathways such as AMPK//SIRT1/PGC1α [131], might lead to an increase of autophagic flux and mitochondrial biogenesis (see [5,131]), driving to the formation of new healthy mitochondria and to the proper cAMP and MAM proteins levels. This pathway could also be linked to Ca^2+^ homeostasis; indeed, in colon cancer cells it has been shown that resveratrol induces a metabolic reprogramming, increasing oxidative capacities, pyruvate dehydrogenase activity, and ATP production. These effects were abrogated by Ca^2+^ chelation or the blockade of the mitochondrial Ca^2+^ uniporter as well as by the inhibition of AMPK pathway [132]. 

## 5. Conclusions

The aim of the present study was to investigate the effect of resveratrol on deregulated cAMP and Ca^2+^ homeostasis in human skin *parkin*-mutant fibroblasts, a *parkin*-null cellular model. The OXPHOS efficiency improvement by resveratrol, via the AMPK/SIRT1/PGC1α pathway, which we reported in *parkin*-mutant fibroblasts in our previous study [5], can be mechanistically linked to three major causes, altered cAMP and Ca^2+^ levels and modulation of protein expression at the ER–mitochondria contact sites. In this study we showed that resveratrol induces a significant increase of cytosolic and mitochondrial Ca^2+^ level in *parkin*-mutant fibroblasts, resulting in a remodulation of the cAMP level. Moreover, resveratrol induces a significant downregulation of the expression level of Miro2 and Mfn2, proteins involved in the ERMCSs, highly expressed in *parkin*-mutant fibroblasts, likely regulating the Ca^2+^ traffic between ER and mitochondria. These findings might shed new light in identifying novel molecular targets for PD treatment.

## Data Availability

Authors declare that data shared are in accordance with consent provided by participants.
